# Linkages between soil, crop, livestock, and human selenium status in Sub‐Saharan Africa: a scoping review

**DOI:** 10.1111/ijfs.15979

**Published:** 2022-08-02

**Authors:** Beaula Mutonhodza, Edward J. M. Joy, Elizabeth H. Bailey, Murray R. Lark, Muneta G. M. Kangara, Martin R. Broadley, Tonderayi M. Matsungo, Prosper Chopera

**Affiliations:** ^1^ Department of Nutrition, Dietetics and Food Sciences University of Zimbabwe P.O. Box MP167, Mt Pleasant Harare Zimbabwe; ^2^ London School for Hygiene and Tropical Medicine Keppel Street London WC1E 7HT UK; ^3^ Rothamsted Research West Common Harpenden AL5 2JQ UK; ^4^ School of Biosciences University of Nottingham Sutton Bonington Campus Loughborough Leicestershire LE12 5RD UK

**Keywords:** Animal tissue, cereals and grains, dietary, nutrition, se deficiency

## Abstract

Selenium (Se) is essential for human health, however, data on population Se status and agriculture‐nutrition‐health linkages are limited in sub‐Saharan Africa (SSA). The scoping review aims to identify linkages between Se in soils/crops, dietary Se intakes, and livestock and human Se status in SSA. Online databases, organisational websites and grey literature were used to identify articles. Articles were screened at title, abstract and full text levels using eligibility criteria. The search yielded 166 articles from which 112 were excluded during abstract screening and 54 full text articles were assessed for eligibility. The scoping review included 34 primary studies published between 1984 and 2021. The studies covered Se concentrations in soils (*n* = 7), crops (*n* = 9), animal tissues (*n* = 2), livestock (*n* = 3), and human Se status (*n* = 15). The evidence showed that soil/crop Se concentrations affected Se concentration in dietary sources, dietary Se intake and biomarkers of Se status. Soil types are a primary driver of human Se status and crop Se concentration correlates positively with biomarkers of Se dietary status. Although data sets of Se concentrations exist across the food system in SSA, there is limited evidence on linkages across the agriculture‐nutrition nexus. Extensive research on Se linkages across the food chain is warranted.

## Graphical abstract

Selenium linkages in Sub‐Saharan Africa.

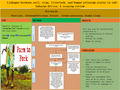

## Introduction

The global burden of malnutrition is unacceptably high and affects every country in the world (UNICEF, 2018). Malnutrition encompasses undernutrition, including micronutrient deficiencies (MNDs), which is often linked to stunting and wasting in children (UNICEF, 2020). Children aged 6–59 months and women of reproductive age (WRA; 15–49 years) are typically the most vulnerable to mineral deficiencies (Kumssa *et al*., 2015; Willett *et al*., 2019). In general, iron (Fe), zinc (Zn), and iodine (I) have been the minerals of greatest public health concern based on global disease burdens in sub‐Saharan Africa (SSA; Wang *et al*., 2016). Although not usually included in national surveys, Se is an essential mineral and widespread deficiencies are likely in SSA (Joy *et al*., 2015a; Ligowe *et al*., 2020b).

Se deficiencies can lead to gestational complications, miscarriages, and damage to the nervous and immune systems of a fetus (Pieczyńska & Grajeta, 2015). Low concentrations of Se in blood serum in the early stage of pregnancy is a predictor of low‐birth weight babies (Pieczyńska & Grajeta, 2015). These factors can predispose populations to inter‐generational malnutrition if not addressed (Imdad *et al*., 2017). Se is a potent antioxidant that plays an important role in inflammatory or immune‐related diseases including anti‐viral immunity, autoimmunity, sepsis, allergic asthma (Huang *et al*., 2012), and metabolic signalling in strokes (Amani *et al*., 2019). Poor quality diets can lead to micronutrient deficiencies, especially in populations with low total food intake (Biesalski & Black, 2016). In SSA, the diets of forty countries consist primarily of carbohydrates (>55% of the overall dietary energy supply (DES) with protein supplying less than 15% of overall DES; Abrahams *et al*., 2011). Carbohydrate rich foods typically have low concentrations of Se compared with protein‐based foods (Combs, 1988; Donovan *et al*., 1991; Joy *et al*., 2015a). Low dietary diversification is an important contributory factor to inadequate human dietary Se intake which consequently leads to human Se deficiency (Gashu *et al*., 2018; Ngigi *et al*., 2020). The World Health Organisation (WHO) recommends a daily intake of 55 μg of Se for adults (WHO, 2016). The Se requirements for pregnant and lactating women are considered to be higher, and range from 60 to 70 μg day^−1^. Requirements for children range between 15 and 40 μg day^−1^ depending on age (NRC and IOM, 2000; Institute of Medicine, 2010).

Dietary Se intake and the Se status of people varies markedly around the world (Fordyce, 2012). This is due to differences in geological, soil geochemical, and climatic factors which result in variation in soil and crop Se status (Fordyce *et al*., 2000; Rayman, 2000; Fairweather‐Tait et al., 2011; Ligowe *et al*., 2020a). Among national food systems in Africa, the prevalence of inadequate dietary Se intake from Se supplies was estimated to be 28% using food composition data from a national and/or regional level (Joy *et al*., 2014). Greater risks of Se deficiency were estimated to occur in the Eastern (52%) and Middle (49%) regions of Africa, followed by Southern (26%), Northern (12%), and Western (6%) regions (Joy *et al*., 2014).

Ligowe *et al*. (2020b) reported circumstantial evidence to support the hypotheses that many soils in SSA provide inadequate Se to food crops for optimal human health, especially where access to animal source foods is limited (Ligowe *et al*., 2020b). However, they concluded that data on Se concentration in soil, crops, livestock, and human biomarkers are generally sparse across SSA. Evidence of the linkages across the agriculture‐nutrition‐health nexus will enable the translation of information across countries and may help inform micronutrient surveillance programs and policy interventions. The aim of this review was therefore to identify linkages between Se in soils/crops, dietary Se concentration, and livestock and human Se status in SSA. The review puts a focus on the Se status of women and children as this group is deemed most vulnerable to micronutrient deficiencies (Kumssa *et al*., 2015; Willett *et al*., 2019) and any nutrition programming targeting this group will have a greater impact on public health outcomes (von Grebmer *et al*., 2014).

## Methods

### Study design

This study is a scoping literature review of mainly published articles and grey literature. The grey literature consisted of project reports and unpublished research theses and data. The inclusion criteria were as follows; Quantitative studies written in, or translated to English were considered with no restrictions on the year of publication. The review featured studies that focused on soil, crop, livestock, women, and children in SSA. The design of the scoping review was carried out according to the 2018 PRISMA Extension for Scoping Reviews (PRISMA‐ScR) checklist which clarifies the purpose and comprehensiveness of the scoping process (Tricco *et al*., 2018).

### Search strategy

Electronic searches for all experimental studies, reviews, meta‐analyses, and reports on Se concentration in soil, crop, livestock, and people in SSA were conducted. Searches were conducted using the following electronic databases: Web of Science, Semantic Scholar, PubMed, Mendeley, and official websites for the United Nations, WHO and the Food and Agricultural Organisation (FAO). Search terms were developed under two headings: Se status of livestock, women and children and Se concentration of soils, crops and animal tissue. Individual livestock names, key words and combinations were used to perform a comprehensive search of the databases, e.g., *Se AND soil AND Sub‐Saharan Africa; Se AND crop AND Sub‐Saharan Africa; Maternal OR mother OR women Se status and Sub‐Saharan Africa; Child OR baby OR children Se status AND Sub‐Saharan Africa; staple diets Sub Saharan Africa; Se and plasma or serum or blood and Sub‐Saharan Africa*. The electronic database was supplemented with scanning of reference lists of relevant reviews. Overlapping data sets were removed using Mendeley *remove duplicate* function. Articles and review articles with dates of coverage spanning from 1972 to 2021 were identified.

### Eligibility

Studies conducted in SSA were included if they reported on Se concentration of soils (*n* = 7 studies), crops (*n* = 9), animal tissues (*n* = 2), livestock (*n* = 3), or human Se status (*n* = 15). Human studies that used whole blood (1), plasma (8), or serum (6) Se as biomarkers of Se status were included but those that used hair and toenails were excluded. Measurement of Se in hair or toenails may provide a useful indication of longer‐term exposure to Se, however due to the lack of widely used thresholds to define Se adequacy or deficiency and the high risk of contamination with exogenous Se from hair products or soil particles these studies were excluded (Phiri *et al*., 2019). Human studies with age and sex dis‐aggregated data were used and data were considered only for apparently healthy children and WRA. For clinical studies, data were only considered for the control groups that did not exhibit clinical symptoms. Soil Se extraction methods were considered for comparability of results, for this study soil data are based on strong acid/peroxide digests. Studies included typically determined Se concentration by inductively coupled plasma mass spectrometry (ICP‐MS; *n* = 26). Other analysis methods used were, atomic absorption spectrophotometry (AAS; *n* = 3) including hydride generation (HG‐AAS; *n* = 4) and electrothermal (EAAS; *n* = 1), inductively coupled plasma atomic emission spectrometry (ICP‐AES; *n* = 1), inductively coupled plasma optical emission spectrometry (ICP‐OES; *n* = 1), spectrofluorimetry (*n* = 1), neutron activation analysis (NAA; *n* = 2), and particle induced X‐ray emission (PIXE; *n* = 1).

### Data extraction

Data were extracted manually into tables in Microsoft Excel to record the required information, including from supplementary material where these were available. Data extracted included: total soil Se concentration, Se concentration of commonly consumed crops and animal source foods in SSA, Se status of livestock, children and WRA, and publication characteristics. Total soil Se concentration data used in this study are all based on acid digests and not being compared with other data based on extractions designed to measure ‘available’ Se or similar. Crop and animal edible tissue Se concentrations were calculated, with dry weight (DW) data converted into wet weight (WW) the form in which the food is consumed using the formula (US‐EPA, 2011):

(1) $$\mathrm{WW}=\mathrm{DW}\times \left[\left(100-\%\text{moisture}\right)/100\right]$$

Moisture content was based on data from a fitted United States Department of Agriculture (USDA) food item (USDA, 2013). Livestock and human Se status as indicated by plasma/whole blood Se concentrations was used. Units of measurement were standardised for comparability purposes and converted to micrograms per litre (μg L^−1^) or micrograms per kilogram (μg kg^−1^). Measures of central tendency, mean and standard of variation (SD) or medians and ranges rounded to the nearest whole number were used to report Se concentrations.

### Outcomes

The primary outcomes were Se concentration in soil, crop, and edible animal tissue, and Se status of livestock, children and WRA. Total soil Se concentration less than 400 μg kg^−1^ was considered low (Fordyce, 2012). Commonly consumed foods with Se concentrations less than 45 μg kg^−1^ WW were regarded as having low Se concentration (Courtman *et al*., 2012). Livestock was considered Se deficient if the plasma Se concentration was less than 30 μg L^−1^ (Mpofu *et al*., 1999) and marginally deficient if less than 60 μg L^−1^ (Dermauw *et al*., 2014). In the human body, deficiency of Se was noted when its amount in plasma was less than 70 μg L^−1^ (Thomson, 2004; Phiri *et al*., 2019; Belay *et al*., 2020).

### Ethical approval

Ethical approval was not required as the study did not have a direct involvement with human participants and the human studies included were based on published data.

### Protocol registration

This protocol was not registered with any health research entity as it is does not report on a health‐related outcome (CRD, 2016).

### Study selection

**Figure 1:**
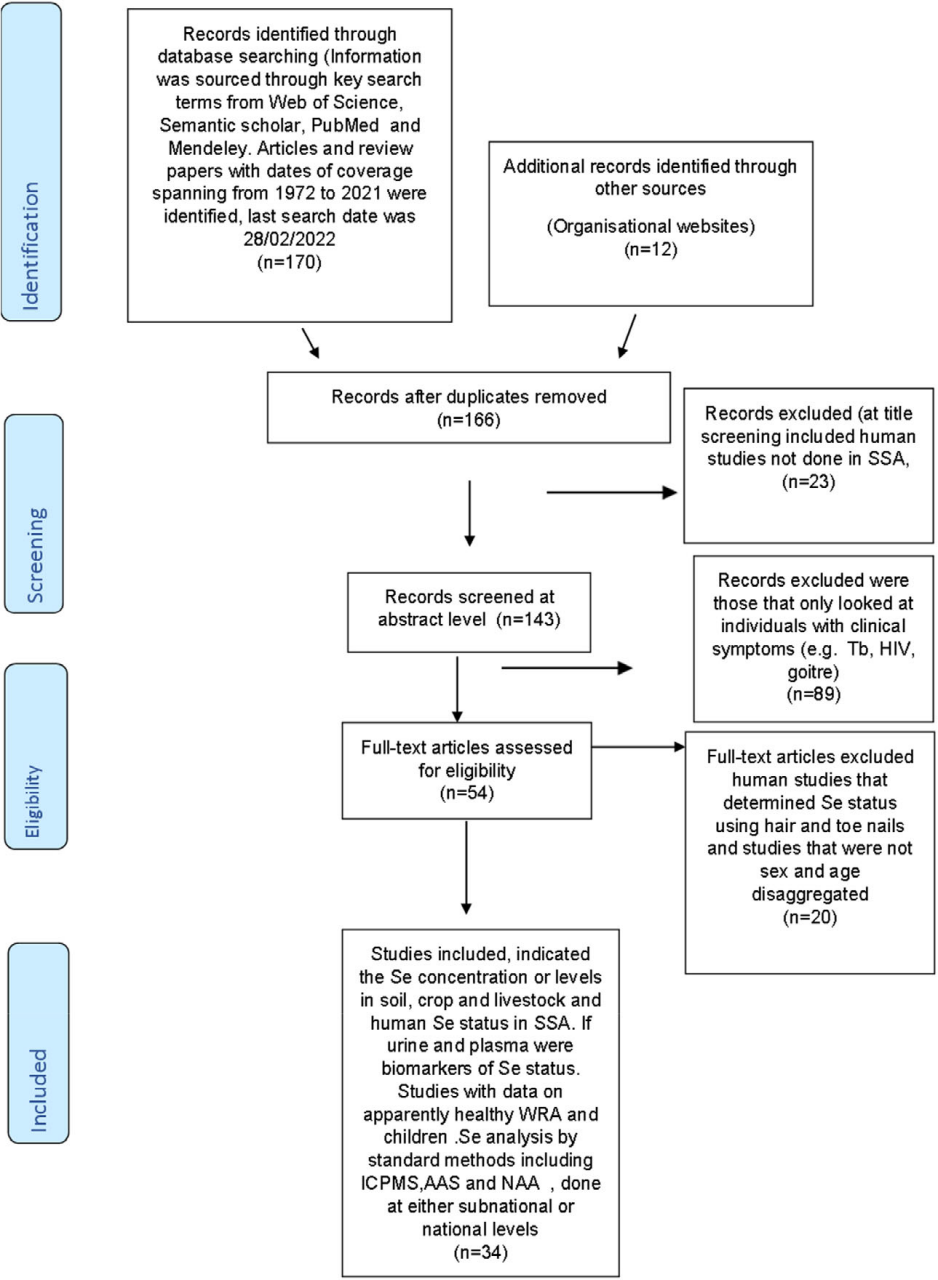
PRISMA study selection. A total of 166 papers were identified upon removal of duplicates, 112 were excluded at title and abstract screening. The remaining 54 full text articles were assessed for eligibility and only 34 papers were included for the scoping review.

A total of 166 articles were identified upon removal of duplicates; 112 were excluded at title and abstract screening. The remaining 54 full text articles were assessed for eligibility and 34 articles were retained for the scoping review (Fig. 1).

## Results

### Total soil se concentration in SSA

Total soil Se data from six SSA countries were identified (Table 1). Among the six countries Zimbabwe, Zambia and Malawi had sub‐optimal (˂400 μg kg^−1^) soil Se concentrations, with the lowest being observed in North east Zimbabwe (Fordyce *et al*., 1994). Kenya had intermediate values of total Se concentration that ranged from 215 to 703 μg kg^−1^ (Ngigi *et al*., 2020). Subjective to the small sample size used compared with the other countries Nigeria and Tanzania had optimal (>600 μg kg^−1^) total soil Se concentrations with Tanzania having the highest maximum total soil Se concentrations.

**Table 1:** Total Soil Se concentration ranges in SSA

Location	Sample description	Soil Se concentration (μg kg^−1^), median (range)	Digestion method	Analytical method	References
Kenya	*n* = 160 8 sites	465 (215–703)	Nitric and hydrofluoric acids	ICP‐MS	Ngigi *et al*. (2020)
Malawi	*n* = 88 10 soil types	162 (50–620)	Nitric, hydrofluoric, and perchloric acids	ICP‐MS	Joy *et al.* (2015b)
Nigeria	*n* = 3 3 zones	1130 (1070–1190)	Hydrochloric, nitric, hydrofluoric, and perchloric acids	AAS	Aremu *et al*. (2010)
Tanzania	*n* = 31 3 soil types	600 (210–4700)	Nitric and hydrofluoric acids	ICP‐MS	Mills & Milewski (2007)
Zambia	*n* = 190 3 zones	70 (trace–790)	Hydrogen peroxide, perchloric, and nitric acids	AAS	Melse‐Boonstra *et al.* (2007)
Zimbabwe	*n* = 70 3 zones	56 (10–163)	Hydrochloric acid, nitric, and perchloric acids	HG‐AAS	Fordyce *et al*. (1994)
Zimbabwe	*n* = 350 2 zones	430 (30–1100)	Hydrochloric acid, nitric, hydrofluoric, and perchloric acids	ICP‐MS	Manzeke‐Kangara MG, unpublished results

ICP‐MS, inductively coupled plasma mass spectrometry; AAS, atomic absorption spectrophotometry; HG‐AAS, hydride generation‐atomic absorption spectrophotometry.

### Crop se concentration in SSA

Maize (*Zea mays*) is the principal staple in most countries within Eastern and Southern Africa (FAO, 2016). Maize grain in Kenya, Malawi, South Africa and Zambia typically had suboptimal Se concentrations (˂45 μg kg^−1^ WW) with median values ranging from 13 to 26 μg kg^−1^ WW (Table 2). However, maize grain from Tanzania (Watts *et al*., 2019) and Uganda (Bevis & Hestrin, 2021) had adequate Se concentration with median values >45 μg kg^−1^ WW. Among the grains; rice (*Oryza sativa*), finger millet (*Eleusine coracana*) and wheat (*Triticum aestivum*) sorghum (*Sorghum bicolor*) had relatively higher median Se concentrations across all countries followed by maize. Roots and tubers are an important constituent of SSA diets, particularly in Central and Western Africa (FAO, 2016). The lowest median Se concentration in cassava (*Manihot esculenta*) was observed in Kenya and the highest in Tanzania. Sweet potatoes (*Ipomoea batatas*) from Malawi had the lowest median Se concentration and Tanzania had the highest. Median Se concentration in beans (*Phaseolus vulgaris*) were optimal (>45 μg kg^−1^ WW) in Tanzania and Uganda and sub‐optimal (˂45 μg kg^−1^ WW) in Kenya and Malawi. Cowpea (*Vigna unguiculata*) Se concentration was highest in Malawi and lowest in Kenya. Generally, Tanzania had higher Se concentration across all crops compared with the other countries.

**Table 2:** Se concentrations in common plant foods in SSA

Crop	Country	Sample description	Se concentration (μg kg^−1^ WW), mean (SD) or median (range)	Analytical method	References
Beans	Kenya	Field (*n* = 130)	33 (4–1280)	ICP‐MS	Watts *et al*. (2019)
	Malawi	Field (*n* = 6)	37 (4–69)	ICP‐MS	Joy *et al*. (2015b)
	Tanzania	Field (*n* = 27)	135 (19–1800)	ICP‐MS	Watts *et al*. (2019)
	Uganda	Field (*n* = 92)	90 (13–3098)	ICP‐MS	Bevis & Hestrin (2021)
Cassava	Kenya	Field (*n* = 18)	4 (2–17)	ICP‐MS	Watts *et al*. (2019)
	Nigeria	Field (*n* = 13)	5 (6)	HG‐AAS	Zarmai *et al*. (2013)
	Tanzania	Field (*n* = 2)	29 (6–51)	ICP‐MS	Watts *et al*. (2019)
	Uganda	Field (*n* = 108)	9 (2–174)	ICP‐MS	Bevis & Hestrin (2021)
Cowpea	Kenya	Field (*n* = 44)	32 (4–308)	ICP‐MS	Watts *et al*. (2019)
	Malawi	Field (*n* = 17)	100 (13–400)	ICP‐MS	Joy *et al*. (2015b)
	Zimbabwe	Field (*n* = 23)	35 (10–88)	ICP‐MS	Manzeke‐Kangara (unpublished results)
Finger millet	Kenya	Field (*n* = 13)	21 (10–557)	ICP‐MS	Watts *et al*. (2019)
	Malawi	Field (*n* = 7)	29 (11–77)	ICP‐MS	Joy *et al*. (2015b)
	Zimbabwe	Field (*n* = 8)	27 (10–79)	ICP‐MS	Manzeke‐Kangara (unpublished results)
Maize	Kenya	Field (*n* = 217)	26 (5–1201)	ICP‐MS	Watts *et al*. (2019)
	Malawi	Field (*n* = 88)	17 (4–478)	ICP‐MS	Chilimba *et al*. (2011)
	Malawi	Field (*n* = 1608)	45 (90)	ICP‐MS	Gashu *et al*. (2021)
	South Africa	Silos (*n* = 896)	13 (0–53)	HG‐AAS	Courtman *et al*. (2012)
	Tanzania	Field (*n* = 42)	143 (5–1187)	ICP‐MS	Watts *et al*. (2019)
	Uganda	Field (*n* = 112)	65 (8–970)	ICP‐MS	Bevis & Hestrin (2021)
	Zambia	Field (*n* = 36) Market (*n* = 31)	14 (8–43)	ICP‐MS	Gondwe (2018)
Rice	Malawi	Field and stores White (*n* = 21) Brown (*n* = 33)	22 (<4–261)	ICP‐MS	Joy *et al*. (2017)
	Tanzania	Field (*n* = 2)	11 (9–13)	ICP‐MS	Watts *et al*. (2019)
Sorghum	Kenya	Field (*n* = 44)	39 (5–726)	ICP‐MS	Watts *et al*. (2019)
	Malawi	Field (*n* = 45)	205 (5–697)	ICP‐MS	Joy *et al*. (2015b)
	Uganda	Field (*n* = 155)	109 (1–2127)	ICP‐MS	Bevis & Hestrin (2021)
	Zimbabwe	Field (*n* = 11)	64 (5–170)	ICP‐MS	Manzeke‐Kangara (unpublished results)
Sweet potato	Kenya	Field (*n* = 19)	3 (1–24)	ICP‐MS	Watts *et al*. (2019)
	Malawi	Field (*n* = 3)	1 (trace–2)	ICP‐MS	Joy *et al*. (2015b)
	Nigeria	Field (*n* = 13)	8 (5)	HG‐AAS	Zarmai *et al*. (2013)
	Tanzania	Field (*n* = 3)	20 (6–35)	ICP‐MS	Watts *et al*. (2019)
	Uganda	Field (*n* = 87)	11 (1–232)	ICP‐MS	Bevis & Hestrin (2021)
Wheat	Ethiopia	Field (*n* = 328)	80 (107)	ICP‐MS	Gashu *et al*. (2021)
	Zambia	Field (*n* = 6)	7 (6–13)	ICP‐MS	Gondwe (2018)

SD, standard deviation; ICP‐MS, inductively coupled plasma mass spectrometry; HG‐AAS, hydride generation‐ atomic absorption spectrophotometry.

### Se concentrations in animal edible tissues

Se is associated with protein in animal tissues (Combs, 1988; Kieliszek, 2019). Generally, cattle in Burundi had higher Se concentrations in both muscle and organ tissue compared with Ethiopia (Table 3). Organ meat had higher Se concentrations compared with muscle meat across all countries. Among the organs kidneys had the greatest Se concentration in both cows and goats. Se concentration of commonly consumed fish in Malawi including catfish (*Clarias gariepinus*), Chambo (*Oreochromis shiranus*), Usipa (*Engraulicypris breianalis*), and Matemba (*Barbus paludinosus*) had the lowest Se concentrations compared with cow and goat meat. Milk had sub‐optimal (<45 μg kg^−1^ WW) Se concentrations. Goat milk had lower Se concentration compared with cow milk. Largely, meat (cow, goat, and fish) had higher Se concentration compared with milk.

**Table 3:** Se concentrations in animal edible tissues in SSA

Country	Meat/milk type	Se concentration (μg kg^−1^ WW), mean (SD) or median (range)	Analytical method	References
Burundi	Cattle (beef) *Abattoir* (*n* = 11)			
	Muscle	169 (110)	HG‐AAS	Benemariya *et al*. (1993)
	Liver	316 (99)		Benemariya *et al*. (1993)
	Heart	335 (40)		Benemariya *et al*. (1993)
	Pancreas	488 (19)		Benemariya *et al*. (1993)
	Spleen	292 (65)		Benemariya *et al*. (1993)
	Kidney	1428 (21)		Benemariya *et al*. (1993)
Ethiopia	Cattle (beef) *Abattoir* (*n* = 60)			
	Liver	180 (154–209)	ICP‐MS and ICP‐OES.	Dermauw *et al*. (2014)
	Kidney	1138 (1019–1256)		Dermauw *et al*. (2014)
	Semitendinosus muscle	88 (55–116)		Dermauw *et al*. (2014)
	Cardiac muscle	175 (140–204)		Dermauw *et al*. (2014)
Burundi	Goat *farm* (*n* = 11)			
	Muscle	103 (70)	HG‐AAS	Benemariya *et al*. (1993)
	Heart	189 (93)		Benemariya *et al*. (1993)
	Liver	427 (349)		Benemariya *et al*. (1993)
	Pancreas	372 (149)		Benemariya *et al*. (1993)
	Spleen	288 (107)		Benemariya *et al*. (1993)
	Kidney	1194 (235)		Benemariya *et al*. (1993)
	Milk (*mature unprocessed)*			
	Cow (*n* = 19)	26 (5)		Benemariya *et al*. (1993)
	Goat (*n* = 5)	23 (5)		Benemariya *et al*. (1993)
	Fish			
Malawi	Catfish (*n* = 4)	250 (5)	NAA	Donovan *et al*. (1991)
	Chambo (*n* = 7)	260 (5)		Donovan *et al*. (1991)
	Usipa, dried (*n* = 10)	730 (2)		Donovan *et al*. (1991)
	Matemba dried (*n* = 10)	480 (6)		Donovan *et al*. (1991)
	Matemba fresh (*n* = 10)	110 (4)		Donovan *et al*. (1991)

μg kg^−1^ WW, micrograms per kilogram Wet Weight; SD, standard deviation; HG‐AAS, hydride generation‐atomic absorption spectrophotometry; ICP‐MS, inductively coupled plasma mass spectrometry; ICP‐OES, inductively coupled plasma optical emission spectrometry; NAA, neutron activation analysis.

### Se status of livestock in SSA

Livestock Se deficiency (<30 μg L^−1^) and marginal deficiency (<60 μg L^−1^) was observed in Ethiopia, South Africa, and Zimbabwe (Table 4). In Zimbabwe, livestock Se deficiency was reported in calves, steers and cows while in Ethiopia marginal deficiency was observed in bulls. In South Africa, 10% of the sheep were marginally deficient (Erasmus, 1984).

**Table 4:** Se status in livestock in SSA

Country	Livestock	Source and sample size	Analytical method	Se concentration (μg L^−1^), mean (SD) or median (range)	Se status	References
Ethiopia	Bull	Abattoir (*n* = 28)	Plasma ICP‐MS and 2ICP‐OES	45 (36–54)	Marginally deficient	Dermauw *et al*. (2014)
South Africa	Sheep	Farm (*n* = 115)	Whole blood NAA	320 (50–750)	Marginally deficient‐adequate	Erasmus (1984)
Zimbabwe	Calves	Farm (*n* = 40)	Plasma ICP‐AES	17 (5)	Deficient	Mpofu *et al*. (1999)
	Cows	Farm (*n* = 40)		17 (5)	Deficient	Mpofu *et al*. (1999)
	Steers	Farm (*n* = 40)		25 (5)	Deficient	Mpofu *et al*. (1999)

SD, standard deviation; ICP‐MS, inductively coupled plasma mass spectrometry; ICP‐OES, inductively coupled plasma optical emission spectrometry; NAA, neutron activation analysis; ICP‐AES, inductively coupled plasma atomic emission spectrometry.

### Prevalence of Se deficiency in children and women in SSA

Inadequate serum/plasma Se concentrations were found in various SSA countries (Ngigi *et al*., 2020; Ligowe *et al*., 2020b). Se deficient status defined as mean serum/plasma Se concentrations less than 70 μg L^−1^ (Phiri *et al*., 2019; Belay *et al*., 2020) was observed in Ethiopia, Democratic Republic of Congo (DRC), Ivory Coast, Malawi, Nigeria, South Africa, Zambia, and Zimbabwe, with mean serum/plasma concentrations among women and children ranging from 29 to 69 μg L^−1^ (Table 5). In northwest Ethiopia, 60% of children were reported to be Se deficient (Amare *et al*., 2012) and in Malawi, 77% (*n* = 348) of children aged between 6 and 23 months were Se deficient (Gebremedhin, 2020). The prevalence of Se deficiency in Zimbabwe was found to be high with almost half of the children (*n* = 269) being Se deficient (Kuona *et al*., 2014).

**Table 5:** Se deficiency in women and children in SSA

Country	Sampling	Age group	Se concentration (μg L^−1^), mean (SD) or median (range)	Analytical method	References
Ethiopia	Simple random (*n* = 100)	54–78 months	63 (26)	Serum ICP‐MS	Amare *et al*. (2012)
Ethiopia	Cluster (*n* = 628)	6–60 months	61 (11–291)	Serum ICP‐MS	Gashu *et al*. (2016)
Ethiopia	Stratified (*n* = 521)	6–59 months	67 (42–95)	Plasma ICP‐MS	Belay *et al*. (2020)
Democratic Republic of Congo (DRC)	Convenience (*n* = 87)	4–17 years	35 (23)	Serum ICP‐MS	Bumoko *et al*. (2015)
Democratic Republic of Congo (DRC)	Convenience (*n* = 109)	15–49 years	40 (22)	Serum spectrofluorimetry	Ngo *et al*. (1997)
Ivory Coast	Cluster (*n* = 47)	18–69 years	29 (17)	Plasma EAAS	Tiahou *et al*. (2004)
Malawi	Convenience (*n* = 54)	Infants (24 weeks)	60 (13)	Plasma ICP‐MS	Flax *et al*. (2014)
Malawi	Cluster (*n* = 494)	Preschool children	61 (25)	Plasma ICP‐MS	Phiri *et al*. (2019)
Malawi	Survey (*n* = 348)	6–23 months	57 (23)	Plasma ICP‐MS	Gebremedhin (2020)
Malawi	Convenience (*n* = 148)	14–45 years	62 (61–65)	Plasma ICP‐MS	Gibson *et al*. (2011a)
Malawi	Convenience (*n* = 49)	17–38 years	67 (53–76)	Plasma and erythrocyte ICP‐MS	Stefanowicz *et al*. (2013)
Niger	Convenience (*n* = 71)	15–49 years	77 (16)	Plasma NAA and PIXE	Cénac *et al*. (1992)
Nigeria	Convenience (*n* = 30)	13–14 years	63 (27)	Serum ICP‐MS	Olopade *et al*. (2009)
South Africa	Convenience (*n* = 111)	47–58 years	68 (58–71)	Plasma AAS	Jaskiewicz *et al*. (1988)
Zambia	Convenience (*n* = 476)	6 months	50 (47–52)	Serum ICP‐MS	Gibson *et al*. (2011b
Zimbabwe	Convenience (*n* = 269)	7–10 years	85 (16)	Serum ICP‐MS	Kuona *et al*. (2014)

SD, standard deviation; ICP‐MS, inductively coupled plasma mass spectrometry; NAA, neutron activation analysis; PIXE, particle induced X‐ray emission; AAS, atomic absorption spectrophotometry.

## Discussion

### Total soil Se concentration and implications on dietary Se supply in SSA

Low total soil Se concentration is common in SSA (Ligowe *et al*., 2020b) and, in some contexts, this may constrain concentrations of Se in crops and livestock feed (Courtman *et al*., 2012). Total Se concentrations might not be a good indicator of how much Se is available to be taken up by a crop or into forage however, it provides an upper bound for the available soil Se. In soils with total Se concentrations <2000 μg kg^−1^, the relationship between total and bioavailable Se was found to be linear (Statwick & Sher, 2017).

The total Se concentration in the soil, however, may not correlate with Se concentration in plants (Fordyce *et al*., 1994; Courtman *et al*., 2012). Se availability to plants is strongly influenced by physio‐chemical factors. Low soil pH, clay soil texture, high organic matter and iron oxyhydroxides reduce Se availability and subsequently Se concentration in food crops (Stroud *et al*., 2010; Gashu *et al*., 2018). The low total soil Se concentrations observed in Zimbabwe could be attributed to the low soil pH observed in Zimbabwean soils (Fordyce *et al*., 1994) which potentially influences dietary Se availability in the human population (Kuona *et al*., 2014). In Malawi, women from villages with acidic soils in the Zombwe Extension Planning Area (EPA) had lower median dietary Se intakes of 6.5 μg day^−1^ and a lower median plasma Se concentration of 53.7 μg L^−1^ (*n* = 60) compared with women in Mikalango EPA where crops were grown on vertisol soils (characteristically with high pH) and their Se intake was eight‐fold higher (median plasma Se concentration 117 μg L^−1^, *n* = 60; Hurst *et al*., 2013).

Soil types are a primary driver of human Se status (Ligowe *et al*., 2020b). Vertisol soil types have been linked with much higher grain Se concentrations (Chilimba *et al*., 2011; Ligowe *et al*., 2020a, 2020b). The higher Se concentrations in crops observed in Tanzania could be attributable to dominance of vertisol soils (Bationo *et al*., 2012) while much lower Se concentrations are observed in the same crops grown in Malawi where vertisol soil type comprise only 0.5% of the land area (Hurst *et al*., 2013).Vertisols are mostly found in semi‐arid and sub‐humid regions of Ethiopia and Tanzania, though they are also scattered throughout much of southern Africa (Hudson, 1987).

Although vertisol soil types are not widely found in SSA they may have a greater capacity to supply Se to crops than do other soils characteristic of southern Africa. The lack of predominant vertisol soil types in SSA points to the need for implementation of strategies that enhance soil‐to‐crop transfer of Se such as, low organic matter and iron oxyhydroxides and high soil pH could improve supply of the micronutrient in SSA. Application of lime is a low‐cost strategy that could be considered, although there is no robust evidence that this strategy would work. However, the enzymes of the antioxidant system that protect cells by eliminating reactive oxygen species (ROS) including catalase, superoxide dismutase, and numerous peroxidases are more efficient in selenite than selenate (Cartes *et al*., 2005; Mora *et al*., 2015). Selenate predominates in alkaline soils and has higher mobility than selenite, which commonly occurs in neutral or acid soils and is easily adsorbed on oxy hydroxides (Kabata‐Pendias, 2010; El‐Sayed *et al*., 2020). On the other hand, Chen *et al*. (2014) observed that Se in the form of selenite caused a generally increased generation of ROS in plants (Chen *et al*., 2014), while the generation of ROS (hydrogen peroxide) was stronger during the interaction of Se in the form of selenate than selenite (Ríos *et al*., 2009). It would be important to know if there might be Se antioxidant losses from increased soil alkalinity that may offset the gains from improved bioavailability of Se. Future studies that explore these linkages and dynamics are essential.

It would also be beneficial to know if soil pH only affects Se status of soil and crop, or if it expands to other micronutrients. If this finding expands to other elements there is a strong case to be made for investment in enhancing soil pH.

### Crop Se concentration and implications on dietary Se intake

Positive relationships exist between the Se concentration of grain and biomarkers of Se dietary status (Gashu *et al*., 2021). Cereals, roots and tubers are primary energy sources across SSA (Joy *et al*., 2014; Food and Agriculture Organisation, 2016; Gondwe, 2018). For example, in Malawi, >50% of dietary energy is derived from maize (Joy *et al*., 2015a) making it a major contributor to dietary Se intake even where grain Se concentrations are low (Chilimba *et al*., 2011). Pulses (beans) also make a large contribution to total protein intake in SSA (Ligowe *et al*., 2020b) and Eastern Africa has a high total intake of 22 kg capita^−1^ (Food and Agriculture Organisation, 2016). Grains and grasses are non‐Se accumulating thus often contain low concentrations of Se on many soil types (Mayland *et al*., 1989; Saha, 2017).

It is therefore apparent that crop‐based foods are not good sources of Se in many soil types, and this could result in low dietary Se intakes in SSA communities typically dependent on plant‐based diets (Donovan *et al*., 1991; Courtman *et al*., 2012; Gashu *et al*., 2020). Hence, a need to increase crop and human Se intake through employment of agronomic biofortification strategies that ensure loading of Se into staple grains through fertilisation. Se plays a key role in the antioxidant systems in plants, studies have shown that application of Se at low doses protect the plants from variety of abiotic stresses such as temperature (Chu *et al*., 2010; Djanaguiraman *et al*., 2010, 2018), drought (Hasanuzzaman *et al*., 2011), desiccation (Pukacka *et al*., 2011), aging (Djanaguiraman *et al*., 2005; Hartikainen, 2005), and metal stress (Kumar *et al*., 2012; Pandey & Gupta, 2015; Ghorai *et al*., 2022). Se, applied at low concentrations, enhances growth and antioxidative capacity of both mono and dicotyledonous plants (Kavalcová *et al*., 2015; Shalaby *et al*., 2017; El‐Sayed *et al*., 2020; Cipriano *et al*., 2022). However, at high Se doses, it acts as pro‐oxidant and causes oxidative stress in plants (Mroczek‐Zdyrska & Wójcik, 2012; Mora *et al*., 2015; Mroczek‐Zdyrska *et al*., 2017). Thus, it is important to have a balance in the amount of Se concentrations added in Se biofortification of crops, as the occurrence of an antioxidant or pro‐oxidative effect depends on the concentration of Se to be used. Food processing methods that improve the bioavailability of nutrients for example fermentation and advocacy for targeted Se fortification of the commonly consumed crop products at population level can be also be employed to improve human Se dietary intake.

### Significance of animal source foods to dietary se supply

A substantial proportion of dietary Se intake in several countries in SSA has been attributed to fish consumption (Donovan *et al*., 1991; Eick *et al*., 2009; Joy *et al*., 2015a; Food and Agriculture Organisation, 2016). In the staple foods of most SSA countries' diets, fish has the highest Se concentration (Donovan *et al*., 1991). Meat and organ consumption form an important contribution to dietary Se supply, as these tissues have a high concentration of Se (Combs, 1988; Donovan *et al*., 1991; Fairweather‐Tait *et al*., 2010; Kieliszek, 2019).Animal consumption is low in SSA where *per capita* meat consumption is approximately 11 kg annum^−1^, a third less than the global average *per capita* meat consumption (Food and Agriculture Organisation, 2016). Milk (cattle and goat) had low Se concentration and yet dairy represents a primary protein source to SSA consumers with fresh dairy products accounting for more than 90% of total dairy consumption (Food and Agriculture Organisation, 2016). In addition, animal milk (from goats and cows) a poor source of Se, is a common complementary food given to infants and young children (Benemariya *et al*., 1993). Se from animal source foods is unlikely to contribute substantially to the diets of most people in SSA, especially among women and children.

However, consumption of organ meat can be advocated for to improve Se supply, as organ meat is deemed affordable to poor populations in most developing countries (van Heerden & Morey, 2014; Bester *et al*., 2018).Organ meat can serve as an alternative Se dense animal source which could potentially improve the Se status of populations. Data on Se concentrations in animal source foods and consequently the direct linkage between animal protein intake, Se intake and Se status are inadequate in SSA. More research is warranted for a wider range of animal products including poultry which accounts for the largest proportion of total meat consumption in SSA (Food and Agriculture Organisation, 2016).

### Livestock Se deficiency and its implication on dietary Se supply in SSA

There is a paucity of studies on livestock Se status in SSA and relationships between trace element concentrations in plasma and edible tissues have not been widely studied. Dietary Se intake by livestock is essential to secure animal health and prevent Se deficiency, and also to increase Se levels in meat, eggs and milk (Combs, 1988; Haug *et al*., 2007). Current findings suggest that Se deficient livestock potentially yield meat and meat products with low Se concentrations (Dermauw *et al*., 2014), which will in turn influence human dietary Se intakes.

The linkages between livestock Se concentration in plasma and edible tissue could be important for human nutrition, as plasma concentrations might form a more practical tool for early evaluation of Se concentrations in meat, essential for optimal human health (Dermauw *et al*., 2014). Se supplementation of livestock through addition of Se to animal feed or Se enrichment of pastures is a promising approach to improve livestock health and productivity, and increase dietary Se intakes (Alfthan *et al*., 2015). However, a direct link between the sensitivity of human Se status with respect to the variation in livestock Se status is yet to be fully established.

### Se deficiency in women and children in SSA

Se deficiency is widespread among women and children in SSA. The vicious cycle of malnutrition can be addressed by interventions that target this group. However, dietary intake and consumption studies in this group have not been widely assessed and there are a few nationally representative surveys documenting human Se status (Gashu *et al*., 2018; Phiri *et al*., 2019; Belay *et al*., 2020). Se speciation significantly affects the potential benefits of this element to mammal health, being the organic Se forms (selenocysteine and/or selenomethionine) the most effective bioavailable Se species for the animal and human nutrition (Thomson, 2004; Fairweather‐Tait *et al*., 2010; Surai *et al*., 2019). There are several factors that can contribute to Se deficiency other than reduced intake. Antagonistic effects exist between Se, vitamins (A, K, and C; Watts, 1994) and other minerals; copper (Cu; Ranches *et al*., 2021), heavy metals, zinc (Zn), sodium (Na), magnesium (Mg), manganese (Mn; Watts, 1994; Thomson, 2004; Stress *et al*., 2014), and iron (Fe; Petkova‐Marinova *et al*., 2017; Larvie *et al*., 2019). Although Se might be flowing adequately in the environment its absorption in the human body might be inhibited. There is a need to review dietary recommendations in light of dietary forms of Se and the Se interactions that influence Se uptake.

## Summary of evidence

In this scoping review, 34 primary studies published between 1984 and 2021, conducted in SSA addressing Se concentrations in soils (*n* = 7 studies), crops (*n* = 9), animal tissues (*n* = 2), livestock (*n* = 3), and human Se status (*n* = 15) were identified. Although independent data sets of Se concentrations exist across the food system in SSA, our findings indicate a paucity of research focusing specifically on the linkages across the agriculture nutrition nexus. However, the evidence gathered showed that Se deficiency is widespread in SSA food systems. Soil characteristics such as soil type were reported to influence soil and crop Se concentrations. For example, vertisol soils in Malawi were positively correlated with crop Se concentration which consequently affected human dietary Se intake and biomarkers of Se status. Crop Se concentration influences animal and human Se status. Although animal source foods are a better source of dietary Se than most crops, they are unlikely to be consumed widely among lower‐income groups. Cereals and legumes are the primary source of dietary Se for most of the population in SSA.

## Limitations

The scoping review had some limitations. To meet the objective of reviewing data for apparently healthy children and WRA, only age and gender dis‐aggregated studies were utilised. As such, the results are not fully representative of all the human Se studies conducted in SSA. Furthermore, due to limited published Se data the scoping review used unpublished results that are not publicly available and articles published beyond the last 30 years.

## Conclusions

There is evidence that the majority of agricultural soils in SSA have low concentrations of plant‐available Se which limits entry of Se into food systems. Crop based foods are a major component of diets across SSA, with low consumption of animal‐source foods that serve as good sources of Se compared with plant‐based foods. Crop and livestock Se concentrations are proxy indicators of dietary Se intake and may reflect human Se status. It is apparent that dietary Se supply is typically low in rural populations of SSA compared with their urban counterparts and that Se deficiency in women and children is widespread in SSA. It is therefore, recommended that strategies be identified and implemented that span the whole food system from soil to crop to humans.

However, in resource limited settings as is the case in most SSA countries appropriate strategies can only be implemented effectively after surveys at national and regional levels have identified hot spots and population groups at risk of Se deficiency. More empirical evidence of linkages across the agriculture–nutrition–health domains is required in SSA, as data remain scarce on livestock Se status, bioavailability of Se in foods and spatial variability of human Se status in SSA.

## Funding information

The authors acknowledge funding from the UK Research and Innovation (UKRI) Global Challenges Research Fund (GCRF) [grant number EP/T015667/1], ‘Translating GeoNutrition: Reducing mineral micronutrient deficiencies (MMNDs) in Zimbabwe’, the ‘GeoNutrition’ project funded by the Bill & Melinda Gates Foundation [BMGF INV‐009129], the University of Nottingham, and the University of Zimbabwe.

## Conflict of interest

The authors declare no conflicts of interest.

## Author contributions

**Beaula Mutonhodza:** Conceptualization (lead); visualization (lead); writing – original draft (lead); writing – review and editing (lead). **Prosper Chopera:** Conceptualization (equal); supervision (lead); validation (lead); visualization (equal); writing – original draft (equal); writing – review and editing (equal). **Tonderayi M. Matsungo:** Conceptualization (equal); supervision (lead); validation (equal); visualization (equal); writing – original draft (equal); writing – review and editing (equal). **Martin R. Broadley:** Conceptualization (supporting); funding acquisition (lead); validation (supporting); visualization (supporting); writing – review and editing (supporting). **Muneta G. M. Kangara:** Data curation (supporting); writing – review and editing (supporting). **Murray R. Lark:** Validation (supporting); visualization (supporting); writing – review and editing (supporting). **Elizabeth H. Bailey:** Validation (supporting); visualization (supporting); writing – review and editing (supporting). **Edward J. M. Joy:** Validation (supporting); visualization (supporting); writing – review and editing (supporting).

## Ethics approval and consent to participate

Not applicable.

### Peer review

The peer review history for this article is available at https://publons.com/publon/10.1111/ijfs.15979.

## Data Availability

Data sharing not applicable to this article as no datasets were generated or analysed during the current study.
